# Vital Signs: Demographic and Substance Use Trends Among Heroin Users — United States, 2002–2013

**Published:** 2015-07-10

**Authors:** Christopher M. Jones, Joseph Logan, R. Matthew Gladden, Michele K. Bohm

**Affiliations:** 1 Office of Public Health Strategy and Analysis, Office of the Commissioner, Food and Drug Administration; 2 Division of Violence Prevention, National Center for Injury Prevention and Control, CDC; 3 Division of Unintentional Injury Prevention, National Center for Injury Prevention and Control, CDC

## Abstract

**Background:**

Heroin use and overdose deaths have increased significantly in the United States. Assessing trends in heroin use among demographic and particular substance-using groups can inform prevention efforts.

**Methods:**

FDA and CDC analyzed data from the National Survey on Drug Use and Health and National Vital Statistics System reported during 2002–2013. Trends in heroin use among demographic and substance using groups were compared for 2002–2004, 2005–2007, 2008–2010, and 2011–2013. A multivariable logistic regression model was used to identify characteristics associated with heroin abuse or dependence.

**Results:**

Annual average rates of past-year heroin use increased from 1.6 per 1,000 persons aged ≥12 years in 2002–2004 to 2.6 per 1,000 in 2011–2013. Rates of heroin abuse or dependence were strongly positively correlated with rates of heroin-related overdose deaths over time. For the combined data years 2011–2013, the odds of past-year heroin abuse or dependence were highest among those with past-year cocaine or opioid pain reliever abuse or dependence.

**Conclusions:**

Heroin use has increased significantly across most demographic groups. The increase in heroin abuse or dependence parallels the increase in heroin-related overdose deaths. Heroin use is occurring in the context of broader poly-substance use.

**Implications for Public Health Practice:**

Further implementation of a comprehensive response that targets the wider range of demographic groups using heroin and addresses the key risk factors for heroin abuse and dependence is needed. Specific response needs include reducing inappropriate prescribing and use of opioids through early identification of persons demonstrating problematic use, stronger prescription drug monitoring programs, and other clinical measures; improving access to, and insurance coverage for, evidence-based substance abuse treatment, including medication-assisted treatment for opioid use disorders; and expanding overdose recognition and response training and access to naloxone to treat opioid pain reliever and heroin overdoses.

## Introduction

During 2002–2013, heroin overdose death rates nearly quadrupled in the United States, from 0.7 deaths to 2.7 deaths per 100,000 population, with a near doubling of the rates from 2011–2013 (1). Data from the National Survey on Drug Use and Health (NSDUH) indicate heroin use, abuse, and dependence have increased in recent years. In 2013, an estimated 517,000 persons reported past-year heroin abuse or dependence, a nearly 150% increase since 2007 (2).

During 2002–2011, rates of heroin initiation were reported to be highest among males, persons aged 18–25 years, non-Hispanic whites, those with an annual household income <$20,000, and those residing in the Northeast (3). However, during this period heroin initiation rates generally increased across most demographic subgroups (3). Most heroin users have a history of nonmedical use of prescription opioid pain relievers (3–5), and an increase in the rate of heroin overdose deaths has occurred concurrently with an epidemic of prescription opioid overdoses.

Although it has been postulated that efforts to curb opioid prescribing, resulting in restricted prescription opioid access, have fueled heroin use and overdose, a recent analysis of 2010–2012 drug overdose deaths in 28 states found that decreases in prescription opioid death rates within a state were not associated with increases in heroin death rates; in fact, increases in heroin overdose death rates were associated with increases in prescription opioid overdose death rates (6). In addition, a study examining trends in opioid pain reliever overdose hospitalizations and heroin overdose hospitalizations between 1993 and 2009 found that increases in opioid pain reliever hospitalizations predicted an increase in heroin overdose hospitalizations in subsequent years (7). Thus, the changing patterns of heroin use and overdose deaths are most likely the result of multiple, and possibly interacting, factors. Moreover, there is a lack of research examining recent trends in the prevalence of other substance use among persons using heroin, especially among the high-risk population of heroin users who meet diagnostic criteria for heroin abuse or dependence.

To improve understanding of current heroin use, abuse, and dependence trends and to identify individual-level risk factors that could help tailor prevention efforts, the Food and Drug Administration (FDA) and CDC examined demographic and substance use, abuse, and dependence trends among heroin users in the United States during 2002–2013.

## Methods

Substance use data are derived from the 2002–2013 NSDUH surveys. The NSDUH is conducted annually by the Substance Abuse and Mental Health Services Administration and provides national- and state-level estimates of the use of illicit drugs, including nonmedical use of certain prescription drugs, alcohol, and tobacco among the civilian, noninstitutionalized population aged ≥12 years (2). NSDUH employs a state-based design with an independent, multistage area probability sample within each state and the District of Columbia (2). For this study, the 2002–2013 NSDUH public use files were combined in four, 3-year time intervals: 1) 2002–2004; 2) 2005–2007; 3) 2008–2010; and 4) 2011–2013.

Past-year nonmedical use of prescription drugs is defined as using prescription drugs without having a prescription, or using prescription drugs only for the experience or feeling it causes, during the 12 months preceding the survey interview. Past-year use of marijuana, cocaine, or heroin is defined as use of the substance in the 12 months preceding the survey interview. Past-year abuse or dependence of specific substances (commonly referred to as addiction) was based on diagnostic criteria contained in the *Diagnostic and Statistical Manual of Mental Disorders, Fourth Edition* (8).

Mortality data from the 2002–2013 Multiple Cause of Death Files from the National Vital Statistics System were analyzed to identify heroin-related drug overdose deaths (9). Heroin-related drug overdose deaths were those assigned an underlying cause of death code of X40–X44 (unintentional), X60–X64 (suicide), X85 (homicide), or Y10–Y14 (undetermined intent) with a contributing cause of death ICD-10 code T40.1 (heroin poisoning) using the *International Classification of Diseases, Tenth Revision* (ICD-10).

First, to assess trends in heroin use in the United States, rates of past-year heroin use per 1,000 persons aged ≥12 years were calculated overall and stratified by sex, age, race/ethnicity, place of residence, annual household income, insurance coverage, and substance use (past-year use of marijuana, cocaine, opioid pain relievers, other psychotherapeutics [tranquilizers, sedatives, and stimulants], and past-month binge drinking) for each study time interval. In addition, the percentage of past-year heroin users who also used at least one other drug in the past year were calculated.

Second, to assess high-risk use of other substances among past-year heroin users, the percentages of past-year heroin users who met diagnostic criteria for past-year alcohol, marijuana, cocaine, or opioid pain reliever abuse or dependence were calculated. All rates are based on U.S Census Bureau population estimates. Two-sided t-tests were used to assess statistically significant differences between 2011–2013 rates and earlier survey year groups. To assess trends, bivariate logistic regression models were applied to test p-values of beta coefficients of the year variable.

Third, to identify individual-level risk factors associated with the subset of past-year heroin users who met diagnostic criteria for heroin abuse or dependence, a multivariable logistic regression model incorporating sex, age group, race/ethnicity group, place of residence, annual household income categories, insurance coverage, and the presence or absence of past-year alcohol, marijuana, cocaine, opioid pain relievers, or other psychotherapeutic abuse or dependence was estimated using the 2011–2013 NSDUH data. Associations were reported as adjusted odds ratios with 95% confidence intervals.

Finally, Pearson’s correlation coefficient (r) was used to assess correlation between the trend in rates of heroin abuse or dependence and heroin-related drug overdose deaths during 2002–2013.

## Results

The weighted interview response rate for the NSDUH during the study period (2002–2013) ranged from 72% to 79% each year. The annual average rate of past-year heroin use in 2011–2013 was 2.6 per 1,000 persons aged ≥12 years (Table 1). This rate was significantly higher than the rates for 2002–2004 (1.6) and 2005–2007 (1.8), and represents a 62.5% increase since 2002–2004. Similarly, the overall rate of people meeting diagnostic criteria for past-year heroin abuse or dependence increased significantly during the study period, from 1.0 per 1,000 to 1.9 per 1,000, which represents a 90.0% increase overall and a 35.7% increase since 2008–2010.

Rates of past-year heroin use were higher among men than women for all time intervals; the rate in 2011–2013 for men was 3.6 per 1,000 compared with 1.6 per 1,000 for women; the gap in rates between men and women narrowed between 2002–2004 and 2011–2013. Both men and women experienced significantly higher heroin use rates during 2011–2013 compared with 2002–2004 and 2005–2007. Among age groups, persons aged 18–25 years experienced the largest increase (108.6%) between 2002–2004 and 2011–2013.

The rate of past-year heroin use among non-Hispanic whites increased 114.3% from 1.4 per 1,000 in 2002–2004 to 3.0 per 1,000 in 2011–2013. Past-year heroin use increased across the three annual household income levels (<$20,000; $20,000–$49,000; ≥$50,000) between 2002–2004 and 2011–2013. Individuals with no health insurance as well as those with private or other insurance experienced statistically significant increases in heroin use rates between 2002–2004 and 2011–2013.

During 2002–2013, past-year heroin use increased among persons reporting past-year use of other substances. The highest rate was consistently found among users of cocaine; during 2011–2013, this rate was 91.5 per 1,000. During the study period, the largest percentage increase, 138.2%, occurred among nonmedical users of opioid pain relievers. In this group, the past-year heroin use rate increased from 17.8 per 1,000 to 42.4 per 1,000, but was still considerably lower than the rate among cocaine users.

**FIGURE 1:**
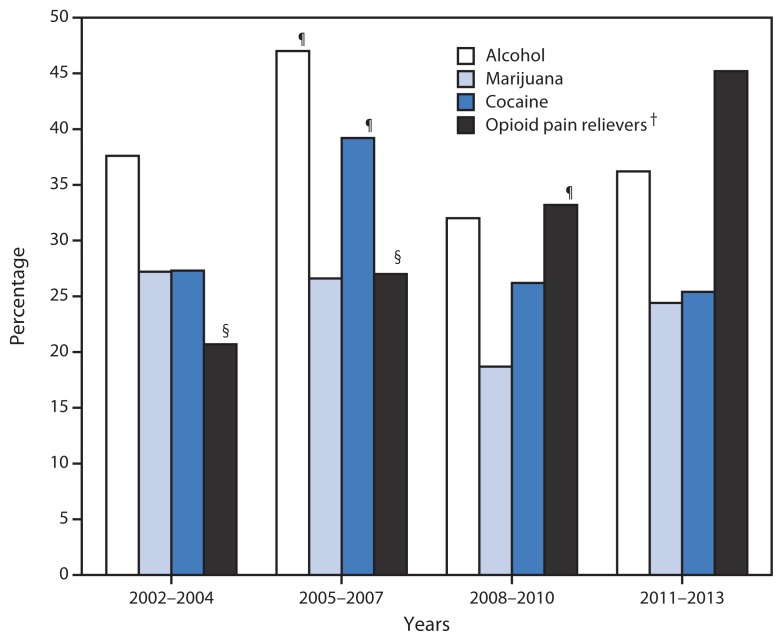
Annual average percentage of past-year heroin users^*^ with past-year selected substance abuse or dependence, by time interval — United States, 2002–2013 ^*^ Past-year heroin use defined as any use of heroin in the 12 months preceding the National Survey on Drug Use and Health survey interview. ^†^ p-value for trend <0.05. ^§^ Rate is statistically significantly different from 2011–2013 rate; p<0.001. ^¶^ Rate is statistically significantly different from 2011–2013 rate; p<0.05.

Overall, 96% of past-year heroin users reported use of at least one other drug during the past year, and 61% reported using at least three different drugs. In addition, a significant percentage of heroin users met diagnostic criteria for past-year abuse of, or dependence on, other substances (Figure 1). The percentage of heroin users with past-year marijuana, cocaine, or alcohol abuse or dependence remained stable during most of the study periods. However, the percentage of heroin users with opioid pain reliever abuse or dependence more than doubled from 20.7% in 2002–2004 to 45.2% in 2011–2013. By 2011–2013, opioid pain reliever abuse or dependence was more common among heroin users than alcohol, marijuana, or cocaine abuse or dependence.

**FIGURE 2:**
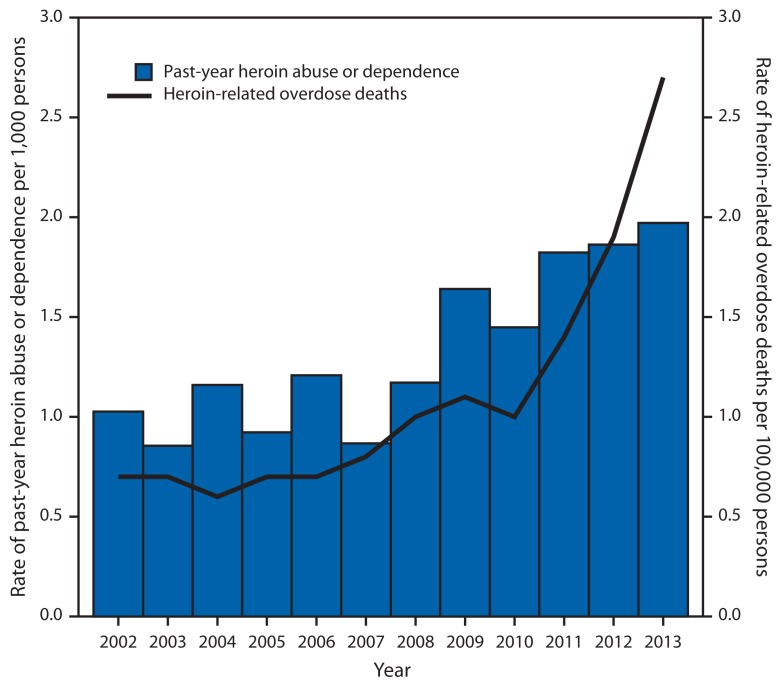
Rates of past-year heroin abuse or dependence^*^ and heroin-related overdose deaths — United States, 2002–2013 ^*^ Past-year heroin abuse or dependence is based on diagnostic criteria contained in the *Diagnostic and Statistical Manual of Mental Disorders, 4th Edition*.

The rate of heroin-related drug overdose deaths was stable at approximately 0.7 per 100,000 during 2002–2006, and began to increase gradually through 2009, when the rate was 1.1 per 100,000. Beginning in 2011, the overdose death rate increased sharply, from 1.4 per 100,000 to 2.7 per 100,000 in 2013, a rate that represents a more than 286% increase since 2002 (Figure 2). There was a strong positive correlation (r = 0.9; p<0.001) between the rates of past-year heroin abuse or dependence and heroin-related drug overdose deaths over time.

The multivariable logistic regression model, adjusted for demographic and specific substance abuse or dependence variables (Table 2), indicates that the following characteristics were associated with higher odds of past-year heroin abuse or dependence: male sex; aged 18–25 years; non-Hispanic white race/ethnicity; residence in a large urban area (Core Based Statistical Area with >1 million persons); <$20,000 annual household income; having no health insurance or having Medicaid; and having past-year abuse or dependence on alcohol, marijuana, cocaine, or opioid pain relievers. Among those with other substance abuse or dependence, the largest adjusted odds ratio (aOR) for heroin abuse or dependence was found among persons with opioid pain reliever abuse or dependence (aOR = 40.0; 95% CI = 24.6–65.3), followed by persons with cocaine abuse or dependence (aOR = 14.7; 95% CI = 7.4–29.2), marijuana abuse or dependence (aOR = 2.6; 95% CI = 1.5–4.6), and alcohol abuse or dependence (aOR = 1.8; 95% CI = 1.2–2.9).

## Conclusions and Comment

There was a significant increase in the rate of past-year heroin use in the United States between 2002–2004 and 2011–2013. Rates remained highest among males, persons aged 18–25 years, persons with annual household incomes <$20,000, persons living in urban areas, and persons with no health insurance or with Medicaid. However, rates increased significantly across almost all study groups. The greatest increases in heroin use occurred in demographic groups that historically have had lower rates of heroin use: doubling among women and more than doubling among non-Hispanic whites. Of particular note is the near doubling in the rate of people with heroin abuse or dependence during the study period, with a 35.7% increase since 2008–2010 alone. This increase parallels the sharp increase in heroin-related overdose deaths reported since 2010.

This study also indicates that the problem of heroin abuse or dependence is not occurring in isolation. Past-year alcohol, marijuana, cocaine, and opioid pain reliever abuse or dependence were each significant risk factors for heroin abuse or dependence. Research has identified poly-substance use as a risk factor for overdose death; most overdose deaths involve multiple drugs (10,11). In 2013, 59% of the 8,257 heroin-related overdose deaths in the United States involved at least one other drug (9). Data presented here indicate the relationship between heroin and opioid pain relievers, as well as the relationship between heroin and cocaine, was particularly strong. In fact, abuse or dependence on opioid pain relievers was the strongest risk factor for heroin abuse or dependence. Taken together, these results underscore the significance of heroin use in the context of broader poly-substance use, a finding that should be considered when prevention policies are being developed and implemented.

The increased availability and lower price of heroin in the United States has been identified as a potential contributor to rising rates of heroin use (12). According to data from the Drug Enforcement Administration’s National Seizure System, the amounts of heroin seized each year at the southwest border of the United States were approximately ≤500 kg during 2000–2008. This amount quadrupled to 2,196 kg in 2013 (12). Since 2010, increased availability of heroin has been accompanied by a decline in price and an increase in purity, which may contribute to its increased use in the United States (13). This increase in the amount of heroin seized, increased availability and purity, and decreased cost are temporally associated with the increases in heroin use, abuse and dependence, and mortality found in this study. Increasing availability points to the importance of public health and law enforcement partnering to comprehensively address this public health crisis.

This study is subject to several limitations. First, NSDUH data are self-reported, and their value depends on the truthfulness and accuracy of individual respondents; under- or over-reporting might occur. Second, because the survey is cross-sectional and different individuals were sampled each year, it is not possible to infer causality from the observed associations. Third, because NSDUH only captures noninstitutionalized civilians, it excludes active duty military personnel, homeless and incarcerated populations, and persons in residential substance abuse treatment programs. Therefore, the drug use estimates in this study might not be generalizable to the total U.S. population, particularly for estimates of uncommonly used drugs like heroin. Finally, the heroin mortality rate is underestimated in the Multiple Cause of Death Files, because the specific drug or drugs involved in the overdose is not specified in approximately 25% of death certificates where the cause of death is drug overdose (14).

These findings indicate significant increases in heroin use across a growing number of demographic groups, including women, the privately insured, and persons with higher incomes. In fact, the gaps in heroin use rates between groups such as men and women, persons with low and higher incomes, and Medicaid and private insurance beneficiaries have narrowed during the past decade. These findings are consistent with recent research documenting significant demographic shifts among people entering heroin addiction treatment over the last 40 to 50 years (4). In addition, persons using heroin are abusing multiple other substances, especially cocaine and opioid pain relievers.

A comprehensive response that targets the wider range of demographic groups using heroin and addresses the key risk factors for heroin abuse and dependence is needed. Specifically, a focus on reducing opioid pain reliever abuse is needed given the strong association between opioid pain relievers and heroin abuse and dependence seen in this study, and prior research indicating that the rate of heroin initiation among people with a history of nonmedical use of opioid pain relievers was approximately 19 times greater than those with no history of nonmedical use (3). Interventions such as prescription drug monitoring programs to reduce inappropriate prescribing of opioids and enable the early identification of persons demonstrating problematic use must be strengthened. The increases in the number of people with heroin abuse or dependence and those dying from heroin-related overdose, as well as the recent increases in hepatitis C virus (HCV) and human immunodeficiency virus (HIV) associated with injection drug use (15,16), underscore the critical importance of improving access to, and insurance coverage for, evidence-based substance abuse treatment. In particular, medication-assisted treatment for opioid use disorders has been shown to reduce opioid use and mortality, and to reduce risk behaviors that transmit HCV and HIV (17). The increases in abuse or dependence and overdose deaths also highlight the urgent need to expand overdose recognition and response training and broaden access to naloxone to treat opioid pain reliever and heroin overdoses.

***Key Points***

- Heroin use in the United States increased 63% from 2002 through 2013. This increase occurred among a broad range of demographics, including men and women, most age groups, and all income levels.
- As heroin use, abuse, and dependence have increased, so have heroin-related overdose deaths. From 2002 through 2013, the rate of heroin-related overdose deaths nearly quadrupled.
- Persons often use heroin with other substances, including marijuana, cocaine, alcohol, and opioid pain relievers. This practice is especially dangerous.
- Groups with an increased risk for heroin abuse or dependence include men, persons aged 18–25 years, non-Hispanic whites, persons with annual household income less than $20,000, Medicaid recipients, and the uninsured.
- States play a key role in addressing heroin use, abuse, dependence, and overdose. States can implement strategies to reduce the abuse of opioid pain relievers, the strongest risk factor for heroin abuse or dependence. They can also improve access and insurance coverage for medication-assisted treatment for opioid use disorders and expand access and training for naloxone administration to reverse overdoses.
- Additional information is available at http://www.cdc.gov/vitalsigns.

## Figures and Tables

**TABLE 1:** Annual average rates* of past-year heroin use† by demographic and substance use characteristics, by time period — United States, 2002–2013

Characteristic	Annual average rate								% change	
	
	2002–2004		2005–2007		2008–2010		2011–2013		2008–2010 to 2011–2013	2002–2004 to 2011–2013
			
	Rate	(95% CI)	Rate	(95% CI)	Rate	(95% CI)	Rate	(95% CI)		
**Overall past-year heroin use**	1.6§§§	(1.4–1.9)	1.8§§	(1.4–2.1)	2.3	(2.0–2.7)	2.6	(2.2–2.9)	13.0	62.5¶
**Overall past-year heroin abuse or dependence**	1.0§§§	(0.8–1.2)	1.0§§§	(0.8–1.3)	1.4§	(1.2–1.7)	1.9	(1.6–2.2)	35.7	90.0¶
**Sex**										
Male	2.4§§	(1.9–2.9)	2.6§	(2.0–3.2)	3.3	(2.6–4.1)	3.6	(3.0–4.3)	9.1	50.0¶
Female	0.8§§	(0.6–1.1)	1.0§	(0.8–1.3)	1.5	(1.2–1.9)	1.6	(1.2–1.9)	6.7	100.0¶
**Age (yrs)**										
12–17	1.8	(1.3–2.5)	1.3	(1.0–1.7)	1.4	(1.0–2.0)	1.6	(1.2–2.2)	14.3	−11.1
18–25	3.5§§§	(2.9–4.3)	4.9§§§	(4.0–5.9)	5.3§	(4.7–6.1)	7.3	(6.4–8.3)	37.7	108.6¶
≥26	1.2§	(1.0–1.6)	1.3	(0.9–1.8)	1.9	(1.6–2.4)	1.9	(1.4–2.4)	0.0	58.3¶
**Race/Ethnicity**										
Non-Hispanic white	1.4§§§	(1.2–1.7)	1.6§§§	(1.3–1.9)	2.6	(2.2–3.0)	3.0	(2.6–3.5)	15.4	114.3¶
Other	2.0	(1.4–2.9)	2.2	(1.5–3.2)	1.9	(1.3–2.7)	1.7	(1.3–2.2)	−10.5	−15.0
**Place of residence**										
CBSA with ≥1 million persons	1.8§§	(1.4–2.2)	2.0§	(1.5–2.6)	2.4	(2.0–2.9)	3.0	(2.4–3.6)	25.0	66.7¶
Other area	1.4§	(1.1–1.8)	1.5§	(1.2–1.9)	2.3	(1.8–2.9)	2.1	(1.7–2.5)	−8.7	50.0¶
**Annual household income**										
<$20,000	3.4§	(2.5–4.6)	3.3§§	(2.4–4.6)	4.4	(3.4–5.7)	5.5	(4.5–6.8)	25.0	61.8¶
$20,000–$49,999	1.3§§	(1.0–1.7)	1.9	(1.5–2.5)	2.7	(2.0–3.6)	2.3	(1.8–3.0)	−17.4	76.9¶
≥$50,000	1.0§	(0.7–1.4)	1.0	(0.6–1.6)	1.4	(1.2–1.7)	1.6	(1.3–1.9)	14.3	60.0¶
**Health insurance coverage**										
None	4.2§	(3.0–5.9)	4.8	(3.6–6.4)	6.3	(4.9–8.0)	6.7	(5.4–8.2)	6.3	59.5¶
Medicaid	4.3	(3.0–6.0)	4.7	(3.1–7.0)	4.3	(3.3–5.6)	4.7	(3.7–5.9)	8.9	9.3
Private or other	0.8§§	(0.7–1.0)	0.8§§	(0.6–1.0)	1.3	(1.0–1.6)	1.3	(1.1–1.6)	0.0	62.5¶
**Substance use**										
Past-month binge drinking	3.7§§	(3.0–4.5)	4.1§	(3.3–5.1)	5.2	(4.3–6.3)	5.8	(4.4–6.4)	11.5	56.8¶
Past-year marijuana use	11.6§§	(9.5–14.1)	13.2	(10.6–16.4)	14.4	(12.6–16.6)	16.9	(14.4–19.8)	17.4	45.7¶
Past-year cocaine use	48.9§§§	(40.2–59.3)	57.6§§§	(45.9–72.2)	68.3§	(55.4–83.9)	91.5	(78.2–106.8)	34.0	87.1¶
Past-year opioid pain reliever nonmedical use	17.8§§§	(14.3–22.0)	25.1§§§	(19.9–31.7)	34.0§	(28.9–39.8)	42.4	(36.6–49.1)	24.7	138.2¶
Past-year other psychotherapeutic nonmedical use**	23.1§§§	(18.6–28.7)	28.5§§§	(23.1–35.1)	41.6	(33.8–51.0)	45.6	(38.9–53.4)	9.6	97.4¶

**Abbreviations:** CBSA = Core Based Statistical Area; CI = confidence interval.

* Rate is per 1,000 population of each analytic group.

† Past-year heroin use is defined as any use of heroin in the 12 months preceding the National Survey on Drug Use and Health survey interview.

§ Rate is statistically significantly different from 2011–2013 rate; ^§^p<0.05;

§§ p<0.01;

§§§ p<0.001.

¶ p-value for trend <0.05.

** Other psychotherapeutics includes tranquilizers, sedatives, and stimulants.

**TABLE 2:** Multivariable logistic regression analysis of demographic and substance use characteristics associated with past-year heroin abuse or dependence* — United States, 2011–2013

Characteristic	Past-year heroin abuse or dependence	

	aOR	(95% CI)
**Sex**		
Male	2.1†††	(1.4–3.0)
Female	1.0	
**Age (yrs)**		
12–17	0.3††	(0.1–0.6)
18–25	1.0	
26	0.6††	(0.4–0.9)
**Race/Ethnicity**		
Non-Hispanic white	3.1†††	(1.8–5.1)
Other	1.0	
**Geography**		
Residing in CBSA with ≥1 million persons	2.4†††	(1.5–3.6)
Residing in other area	1.0	
**Household income (annual)**		
<20,000	1.0	
$20,000–$49,999	0.5††	(0.3–0.7)
≥$50,000 or more	0.6†	(0.3–0.9)
**Insurance coverage**		
None	3.1†††	(2.2–4.3)
Medicaid	3.2†††	(1.9–5.4)
Private or other	1.0	
**Past-year substance abuse or dependence** §		
Alcohol	1.8††	(1.2–2.9)
Marijuana	2.6††	(1.5–4.6)
Cocaine	14.7†††	(7.4–29.2)
Opioid pain relievers	40.0†††	(24.6–65.3)
Other psychotherapeutics¶	1.6	(0.8–3.2)

**Abbreviations:** aOR = adjusted odds ratio; CBSA = Core Based Statistical Area; CI = confidence interval.

* Past-year heroin abuse or dependence is based on diagnostic criteria contained in the *Diagnostic and Statistical Manual of Mental Disorders, 4th Edition*.

† Statistically significant finding; ^†^p<0.05;

†† p<0.01;

††† p<0.001.

§ Referent group is no past-year abuse or dependence.

¶ Other psychotherapeutics includes tranquilizers, sedatives, and stimulants.
